# Impact of functional evaluation 3D software on impingement and clinical outcomes in robotic-arm assisted total hip arthroplasty

**DOI:** 10.1186/s42836-025-00332-z

**Published:** 2025-09-26

**Authors:** Enrico Bertugli, Andrea Marcovigi, Filippo Selleri, Gianluca Grandi, Francesco Zambianchi, Fabio Catani

**Affiliations:** 1 Department of Orthopaedic Surgery, Azienda Ospedaliero-Universitaria Policlinico Di Modena, 41125 Modena, Italy; 2 University of Modena and Reggio-Emilia, 41125 Modena, Italy

**Keywords:** THA, MAKO, Spine, Impingement, 3D, PROMs, FJS, HOOS, OHS, Robotic

## Abstract

**Background:**

This study aims to investigate whether individualized component positioning in robotic-assisted total hip arthroplasty (RTHA) using three-dimensional (3D) Functional Component Positioning Assessment (FCPA) reduces prosthetic and bone impingement and improves Patient-Reported Outcome Measures (PROMs).

**Methods:**

This retrospective study includes 100 consecutive patients who underwent RTHA at a single center between May 2021 and March 2022 using the Mako THA system. Preoperative imaging and planning incorporated sacral slope (SS) measurements. SS values in standing and sitting positions were uploaded into the robotic software to simulate prosthetic hip motion during FCPA and guide component orientation. The primary outcome measures included intraoperative impingement analyses using FCPA and postoperative PROMs, including the Oxford Hip Score (OHS), Forgotten Joint Score (FJS-12), and Hip Disability and Osteoarthritis Outcome Score (HOOS).

**Results:**

Impingement was detected in 46% of patients during daily living activities, with prosthesis-on-prosthesis being the most frequent impingement type. No statistically significant differences in PROMs were observed between patients with and without impingement. Nonetheless, overall PROMs were excellent, with a mean HOOS of 94.2 ± 8.3, mean FJS-12 of 87.8 ± 17.2, and mean OHS of 92.3 ± 10. Multivariate analysis revealed that higher BMI negatively impacted PROMs, and the SS difference correlated significantly with OHS and FJS-12 scores (*P* < 0.0001).

**Conclusions:**

Individualized component positioning, even when aided by 3D FCPA, does not eliminate the risk of impingement. The concept of a universal “safe zone” may be inadequate to prevent impingement, emphasizing the need for patient-specific implant positioning strategies based on spinopelvic mobility. While impingement did not significantly impact PROMs, optimizing implant positioning through robotic-assisted techniques may contribute to improved long-term outcomes and reduced dislocation risk.

## Introduction

Total hip arthroplasty (THA) has been called the operation of the century and has proven to be a successful and long-lasting operative procedure with great patient satisfaction and survival rates over 90% after 15 years [1, 2]. However, there is still room for improvement [3]. Hip dislocation remains a nagging cause of early failure. Even when the components are placed accurately within the historically known static safe zones [4, 5], dislocations continue to occur [6].

The interaction and movements of the hip, pelvis, and spine have received great attention in recent years. Degenerative changes of the hip and spine often coexist and may be interrelated, coined the term hip-spine syndrome [2, 7]. Hence, the optimum THA component position algorithm should consider not only acetabular component orientation and femoral stem position, but also the influence of spine movement. Dynamic change in acetabular component orientation during activity has been described as the “functional” acetabular component orientation [2, 8]. Acetabular functional orientation (guided by spinopelvic movement) and stem position should therefore be analyzed in relation to activities of daily living (ADL) to define the impingement-free range of motion (ROM) [9].

Robotic Arm-Assisted THA (RTHA) has been associated with a lower risk of dislocation [10]. Recent robotic software developments allow Functional Component Positioning Assessment (FCPA) for impingement with a computed tomography (CT) based three-dimensional (3D) model of the hip [9], incorporating information related to spinopelvic mobility, and can define an impingement-free safe zone. Using a virtual hip ROM scoring system, O’Connor et al. (2021) [9] identified a combined anteversion safe zone, with the pelvis in standing (30°–50°) and sitting position (45°–65°) that seems to provide maximum impingement-free ROM values.

While the individualized position of the acetabular component, based on individual spino-pelvic mobility patterns, has been demonstrated to reduce the incidence of impingement compared to common, non-individualized positions [2, 11, 12], there is still a lack of knowledge regarding the clinical impact of impingement in THA.

Therefore, this study hypothesized that individualized component positioning through FCPA evaluation leads to an improvement in patient-reported outcome measures (PROMs) due to the reduction of prosthetic and bone impingement following RHTA.

## Materials and methods

### Study design and patient selection

**Fig. 1:**
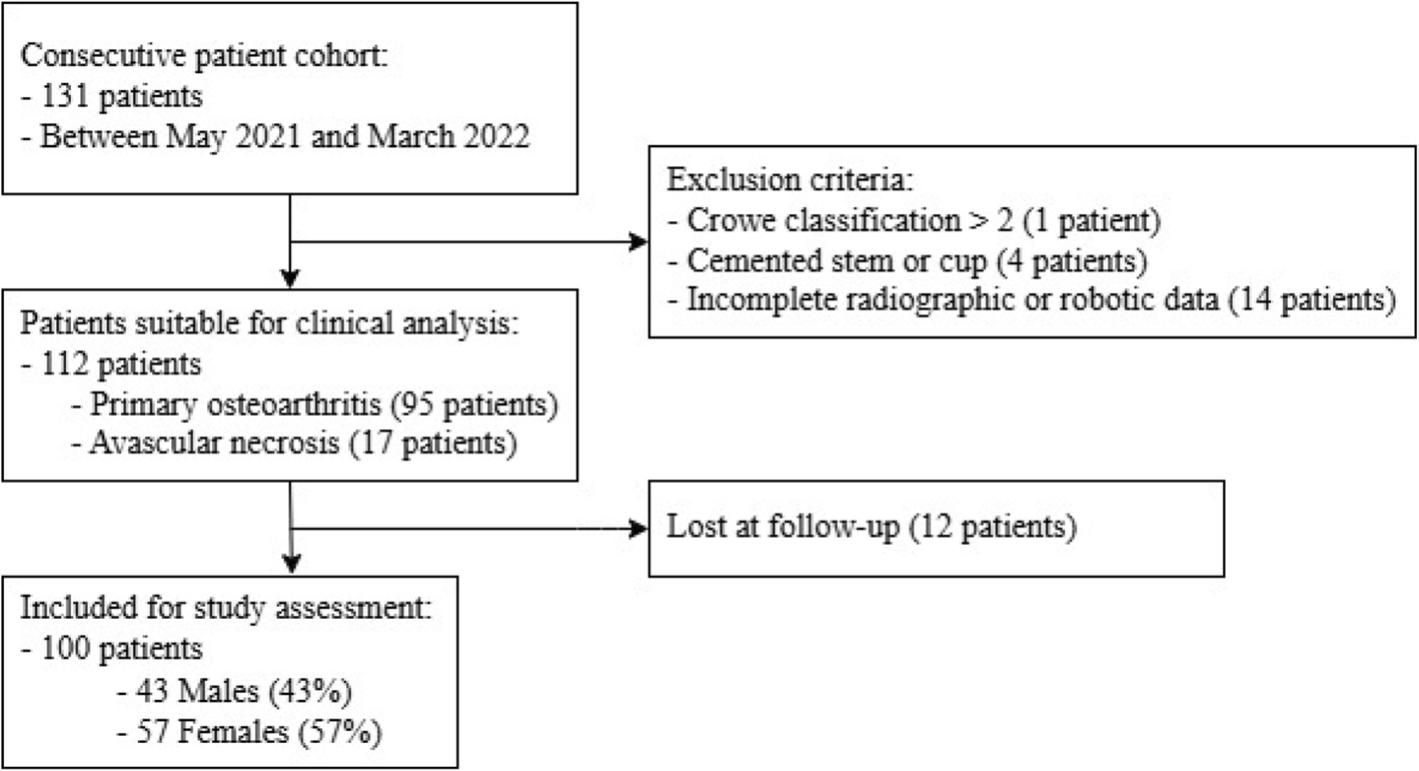
Flowchart of the study cohortF

This retrospective study analyzed 100 consecutive patients who underwent RTHA at a single center between May 2021 and March 2022 using the Mako THA system (Mako Surgical Corp., [Stryker], Fort Lauderdale, Florida). All surgeries were performed by two experienced surgeons, each with an annual caseload of over 80 RTHAs consistently maintained over the past ten years in the same facility. Patients were excluded if they presented with congenital hip dysplasia classified as Crowe grade > 2, were younger than 18 years old, or required the use of a cemented stem or cup. Moreover, patients were excluded if they underwent previous spine surgery or had incomplete radiographic and/or robotic data (Fig. 1).

### Surgical technique

All procedures were conducted by two experienced surgeons using a posterior-lateral approach with patients positioned in lateral decubitus. A piriformis-sparing technique was performed, along with a femur-first approach. The robotic system’s “femur-enhanced workflow” was utilized in all surgeries, providing real-time intraoperative data on broach version, combined anteversion (CoV), leg length, and combined offset. The cementless Accolade® II stem and cementless Trident® II Tritanium® cup (Stryker, Mahwah, NJ, USA) were implanted in all cases.

### Imaging and preoperative planning

Preoperative CT imaging of the lower extremities was performed according to the system’s protocol. Lateral pelvic radiographs were obtained with patients in standing and sitting positions (hip flexion 90°, knee flexion 90°).

Radiographic sacral slope (SS) was measured and defined as the angle between a line tangent to the superior S1 endplate and a horizontal line [13]. Based on the SS difference between standing and sitting positions, patients were classified into three groups: stiff (< 10°), normal (10°–30°), and hypermobile (> 30°) [14].

Sacral slope values in standing and sitting positions were uploaded into the robotic software to guide pelvic and acetabular component orientation during FCPA. In addition to SS, to ensure greater accuracy and reproducibility of the 3D simulation, the system takes into account the anterior pelvic plane (APP), defined as the plane passing through the pubic tubercle and the anterior superior iliac spines [15]. This virtual 3D tool enabled surgeons to simulate prosthetic hip motion in flexion/extension, adduction/abduction, and internal/external rotation. Cup inclination and version variations during pelvic tilting from sitting to standing positions were also assessed, thereby identifying potential preoperative impingement during hip motion. Initial acetabular component positioning was set at 40° inclination and 20° anteversion. Adjustments were made as needed: cup inclination was increased by up to 5° to address superior acetabular uncoverage, and anteversion was adjusted between 15° and 25° to address posterior acetabular coverage. The femoral stem was initially planned with an anteversion angle close to 15° [16], compatible with meta-epiphyseal femoral version [17]. As the target of 15° of stem anteversion cannot always be achieved [18], in a femur-first technique, cup positioning can be modified to optimize CoV. Combined anteversion was calculated as the sum of stem and cup anteversion, to reach the “safe zone” of 25°–50° [4, 19].

**Fig. 2:**
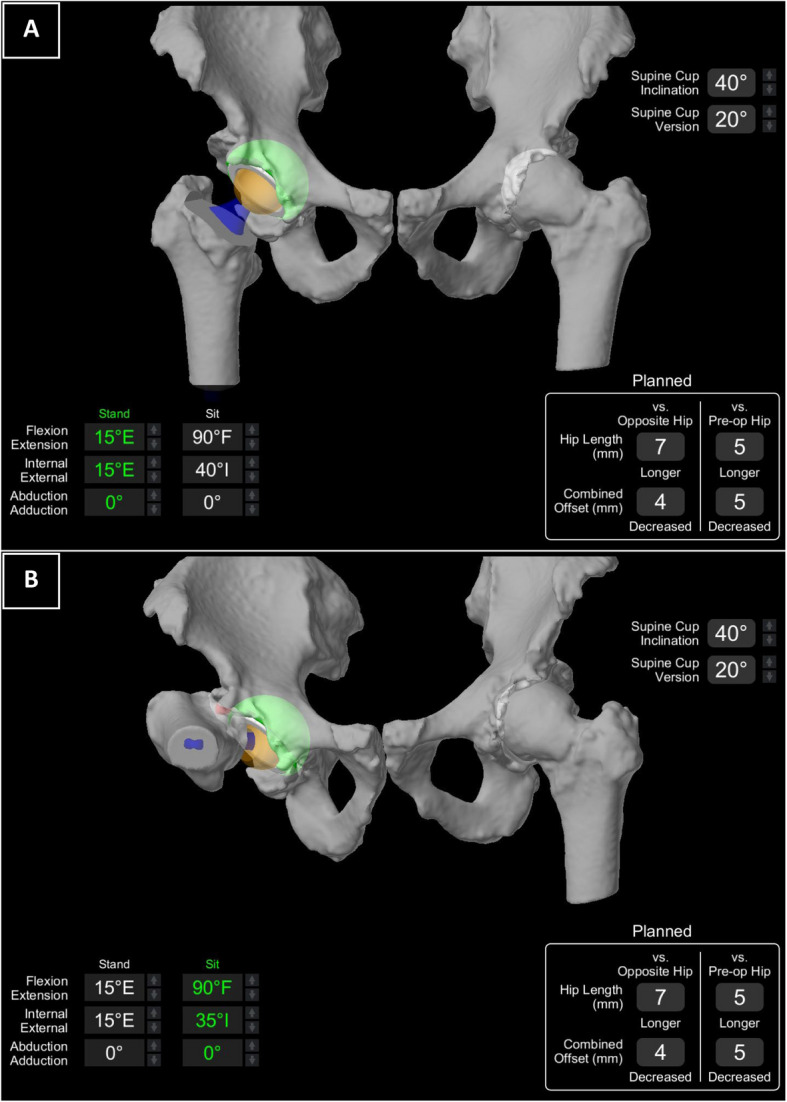
Preoperative FCPA impingement analyses

Cup and stem anteversion were subsequently refined based on impingement analyses performed through FCPA. Through this 3D simulation system, any contact between prosthetic components, bony structures, or between the prosthesis and bone was detected and visually highlighted in red at the point of contact (Fig. 2B). The objective was to prevent any bony or prosthetic impingement in two specific extreme hip positions (Fig. 2), which are expected to encompass the majority of ADLs:

1. Standing Position: Pelvis aligned with the hip in 15° extension, 15° external rotation, and neutral abduction.
2. Sitting Position: Pelvis aligned with the hip in 90° flexion, 35° internal rotation, and neutral abduction.

When impingement could not be reduced by solely modifying the cup and stem version, adjustments were made to increase the offset or leg length. This was achieved by using a varus stem (127°) or modifying the femoral head length, while ensuring adequate leg length discrepancy. Adjustments were made to eliminate any impingement in the two positions mentioned above, with only osteophytic impingement being left in anticipation of intraoperative osteophyte removal (Fig. 2B).

#### Postoperative analysis and data collection

Final implant positions were recorded and collected. Postoperatively, FCPA was used to simulate potential impingements during five routine motor activities of daily living using the data of final implant positioning.

Five motor tasks were evaluated based on the kinematic manoeuvres proposed by Patel et al. [20]:

1. Maximum Flexion: Hip flexion without rotation or abduction in a sitting pelvis position, up to 150° or until impingement occurs.
2. Maximum Extension: Hip extension without rotation or abduction in a standing pelvis position, up to the point of impingement.
3. Rising from a Low Seat: Hip flexion at 100°, abduction at 5°, and internal rotation at 15° with the pelvis in a sitting position.
4. Shoe Tying: Hip flexion at 95°, adduction at 5°, and internal rotation at 15° with the pelvis in a sitting position.
5. Rolling Over: Hip extension at 5°, adduction at 5°, and external rotation at 30° with the pelvis in a standing position.

Impingement types were classified as follows:

- Prosthesis-on-Prosthesis (PP): Contact between the stem neck and liner.
- Prosthesis-on-Bone (PB): Contact between the stem neck and pelvic bone.
- Bone-on-Bone (BB): Contact between femoral and pelvic bones.

If multiple impingement types occurred simultaneously, only the type closest to the center of rotation was recorded (PP > PB > BB). This selection method was applied only in cases where impingement occurred exclusively at a specific degree of movement, with no impingement observed at lower degrees.

### Patient-reported outcome measures (PROMs)

The Oxford Hip Score (OHS), Forgotten Joint Score (FJS-12) [21, 22] and Hip disability and Osteoarthritis Outcome Score (HOOS) [23] were administered pre-operatively and at a minimum follow-up of two years. The Patient-Acceptable Symptom State (PASS) represents the minimum threshold at which a patient perceives their health status as satisfactory. The PASS thresholds have been established at 76.7 points for the HOOS at the 2-year follow-up [24] and 69 points for the FJS-12 at the same time point [21]. The OHS is a concise 12-item questionnaire resulting in a total score ranging from 0 to 48 [25]. In our case series, the OHS was adapted to a 100-point scale to facilitate comparison with other outcome measures. Consequently, no PASS threshold described in the literature can be considered reliable for this modified scoring system.

### Statistical analysis

Continuous variables were expressed using mean and standard deviation; binary and categorical data were reported as frequencies and percentages. The associations among maximum extension and flexion without impingement and the patients’ characteristics were assessed using linear regression models, whereas logistic regression models were adopted to investigate the associations with respect to the presence of impingement in the other three motor tasks. For each outcome, in the first place, we performed univariable analyses and then estimated a final multivariable model. Results were reported as the mean difference (MD) or odds ratio (OR) with 95% confidence intervals (CI). Analyses were carried out using R 4.1.1 statistical software.

## Results

A total of 100 RTHAs were included for assessment. Demographic data of the study cohort are reported in Table 1. The results were presented by categorizing the findings according to spinopelvic mobility, FPCA data, and PROMs.

**Table 1:** Demographic data of the study cohort

Variable	Value
Patients number	100
Women (%)/Men (%)	57 (57%)/43 (43%)
Body Mass Index (m ± sd) [range]	28 ± 5 [19.6–43.8]
Age (years) (m ± sd) [range]	69.9 ± 10.1 [32–89]
Follow up (months) (m ± sd) [range]	31.8 ± 3.2 [26–37]

A total of 100 RTHAs were included for assessment, with a follow-up of at least 2 years

### Spinopelvic mobility and components positioning data

The mean SS values in standing and sitting positions, analyzed by subgroup, are presented in Table 2. The overall mean SS difference between standing and sitting positions was 18.2° (SD 11.4°, range − 9° to 50°). We reported three cases in which anterior pelvic tilt occurred during the sit-to-stand transition (SS difference: − 9, − 2, and − 2).

**Table 2:** Sacral Slope (SS) and Combined Version (CoV) values compared between each subgroup

	**Stiff** **(20%)**	**Normal** **(63%)**	**Hypermobile (17%)**	**Overall** **(100%)**
SS sitting (°)	31.9 ± 5 [10–45]	21.3 ± 9.6 [4–43]	9.9 ± 7.4 [0–30]	21.5 ± 11.5 [0–45]
SS standing (°)	34.9 ± 13.2 [13–54]	39.7 ± 9.8 [19–68]	45.7 ± 7.9 [35–66]	39.7 ± 10.7 [13–68]
Δ SS (°) (stand-sit)	3 ± 5.7 [− 9–9]	18.3 ± 5.9 [10–30]	35.8 ± 4 [31–50]	18.2 ± 11.4 [− 9–50]
CoV standing (°)	40.8 ± 5.3 [30–50]	37.8 ± 8.1 [14–59]	35.7 ± 8 [25–56]	38 ± 7.7 [14–59]
CoV sitting (°)	42.9 ± 5.6 [36–52]	50.2 ± 8.5 [28–74]	58.7 ± 7.8 [49–79]	50.2 ± 9.2 [28–79]
Δ CoV (°) (sit-stand)	2.1 ± 4.1 [− 7–7]	12.5 ± 4.7 [7–23]	22.9 ± 2.5 [19–29]	12.2 ± 7.6 [− 7–29]

Data are presented as mean, standard deviation, and range (brackets). Approximately two-thirds of patients fall into the “normal spine” subgroup, while the remaining one-third are evenly distributed between the “stiff” and “hypermobile” subgroups

**Fig. 3:**
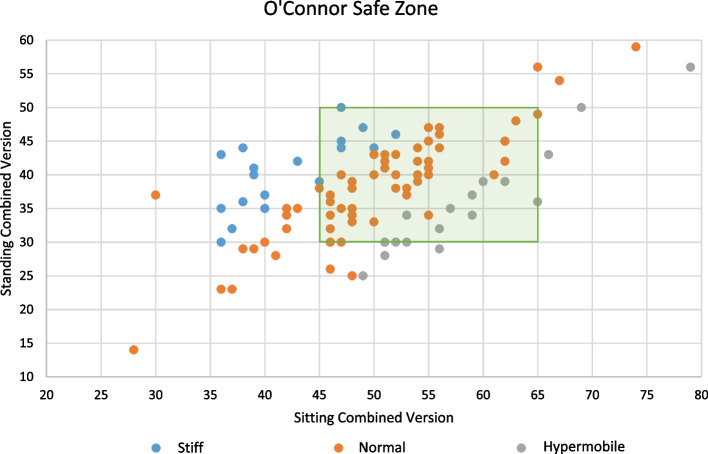
Implant positioning distribution compared to the safe zone of O’Connor

Implant positioning was compared to the functional safe zone proposed by O’Connor. Overall, 66 patients (66%) had FCPA-determined implant positioning (CoV) within the O’Connor Safe Zone. Subgroup analysis revealed that 45% (9/20) of implants in patients with a stiff pelvis were implanted within the safe zone. In contrast, 73% (46/63) of implants in patients with a normal pelvis and 64.7% (11/17) in hypermobile patients were positioned within the safe zone (Fig. 3).

### Descriptive postoperative FCPA data

A detailed breakdown of impingement data according to spinopelvic mobility is presented in Table 3. Analyzing daily living activities (Motor Tasks #3, #4, and #5), it was found that 54% of patients exhibited no impingement. In 5% of cases, impingement was observed in both Motor Task #1 and #2. Only one patient (1%), characterized by a stiff spine, experienced impingement in all movements. In the remaining 40% of patients, impingement occurred in just one motor task (34% of patients experienced impingement exclusively in extension).

**Table 3:** Detailed impingement data according to spinopelvic mobility

FCPA Data	Spinopelvic group	Mean Values	No impingement	Bone on Bone	Prosthesis Bone	Prosthesis on Prosthesis
Maximum Flexion Sitting	Stiff	116.5 ± 13 [95–140]	0 (0%)	14 (70%)	5 (25%)	1 (5%)
	Normal	124 ± 17.2 [55–150]	4 (6.3%)	50 (79.5%)	5 (7.9%)	4 (6.3%)
	Hypermobile	140.3 ± 10.8 [110–150]	3 (17.6%)	12 (70.6%)	1 (5.9%)	1 (5.9%)
Maximum Extension Standing	Stiff	36 ± 10.2 [15–55]	0 (0%)	0 (0%)	1 (5%)	19 (95%)
	Normal	41 ± 11.4 [20–80]	0 (0%)	1 (1.6%)	7 (11.1%)	55 (87.3%)
	Hypermobile	42.7 ± 11.7 [20–65]	0 (0%)	1 (5.9%)	1 (5.9%)	15 (88.2%)
Sit to stand (Flex 100, Abd 5, Ir 15)	Stiff		18 (90%)	1 (5%)	1 (5%)	0 (0%)
	Normal		54 (85.7%)	6 (9.5%)	2 (3.2%)	1 (1.6%)
	Hypermobile		17 (100%)	0 (0%)	0 (0%)	0 (0%)
Shoe Tying (Flex 90, Add 15, Ir 15)	Stiff		19 (95%)	1 (5%)	0 (0%)	0 (0%)
	Normal		57 (90.5%)	5 (7.9%)	1 (1.6%)	0 (0%)
	Hypermobile		17 (100%)	0 (0%)	0 (0%)	0 (0%)
Rolling over (Ext 5, Add 5, Er 30)	Stiff		10 (50%)	3 (15%)	1 (5%)	6 (30%)
	Normal		42 (66.7%)	4 (6.3%)	6 (9.5%)	11 (17.5%)
	Hypermobile		12 (70.6%)	0 (0%)	3 (17.6%)	2 (11.8%)

Analyzing daily living activities (Motor Tasks #3, #4, and #5), approximately one-third of patients experienced impingement exclusively in extension. Flex: flexion, Ext: extension, Abd: abduction, Add: adduction, Ir: internal rotation, Er: external rotation

### Patient-reported outcome measures (PROMs)

Every PROM was administered pre-operatively and at a minimum follow-up of two years, in particular 31.8 ± 3.2 months (range 26–37).

Although PROMs were, on average, slightly higher in patients without impingement, no statistically significant differences were observed (Table 4). No complications, including dislocations, were recorded.

**Table 4:** PROMs analysis in patients with and without impingement

PROMs	Impingement (46%)	No impingement (54%)	*P*-Value (< 0.05)
HOOS postop	94 ± 9.4	94.5 ± 6.8	0.80
Δ HOOS	50.1 ± 16.9	49.1 ± 15.6	0.76
FJS-12 postop	86.3 ± 21.5	89.7 ± 10.2	0.30
Δ FJS-12	56.8 ± 25.2	62.1 ± 18.4	0.23
OHS postop	91.4 ± 12.2	93.4 ± 6.6	0.31
Δ OHS	67.8 ± 14	70.4 ± 10.4	0.29

Although PROMs were, on average, slightly higher in patients without impingement, no statistically significant differences were observed. Postop: postoperative, Δ: delta, difference between postoperative and preoperative

The mean HOOS was 94.2 (SD 8.3), the mean FJS-12 was 87.8 (SD 17.2), and the mean OHS was 92.3 (SD 10). The HOOS PASS threshold (76.7) was not achieved in 8 cases (8%): 2/20 patients (10%) in the stiff spine group, 3/63 (4.8%) in the normal mobility group, and 3/17 (17.6%) in the hypermobile group. Similarly, the FJS-12 PASS threshold (69) was not reached in 8 cases (8%): 5/63 patients (7.9%) in the stiff spine group, and 3/17 (17.6%) in the hypermobile group.

A statistically significant association was identified between high BMI and PROMs at the 2-year follow-up in the univariate analysis (*P* < 0.05). Higher BMI values were associated with poorer postoperative PROMs in four distinct associations (*P* = 0.047, 0.045, 0.045, and 0.048) (Table 5). In the multivariate models, a statistically significant correlation was also found between postoperative OHS and FJS-12 and the increase of delta SS with *P* < 0.000 (Table 5).

**Table 5:** Multivariate analysis on postoperative clinical outcomes

Outcome	Variable	Mean Difference	95% CI		*P*-value (< 0.05)
FJS-12 Post	Age	− 0.36	− 0.78	0.07	0.105
	Gender	4.09	− 4.52	12.70	0.355
	BMI	− 0.68	− 1.35	− 0.02	**0.047**
	Motor Task #1	− 0.01	− 0.25	0.23	0.933
	Age	− 0.37	− 0.79	0.06	0.097
	Gender	4.27	− 4.34	12.87	0.334
	BMI	− 0.69	− 1.35	− 0.03	**0.045**
	Motor Task #2	− 0.09	− 0.44	0.26	0.599
	Age	− 0.06	− 0.25	0.14	0.587
	Gender	− 0.26	− 4.23	3.71	0.898
	BMI	− 0.24	− 0.55	0.07	0.134
	Δ SS (stand-sit)	− 1.13	− 1.27	− 1.00	**0.000**
	Age	− 0.36	− 0.79	0.06	0.100
	Gender	3.92	− 4.68	12.51	0.375
	BMI	− 0.69	− 1.35	− 0.03	**0.045**
	CoV Stand	0.13	− 0.39	0.64	0.638
	Age	− 0.38	− 0.80	0.05	0.084
	Gender	3.91	− 4.62	12.43	0.372
	BMI	− 0.68	− 1.33	− 0.02	**0.048**
	Leg length difference	0.53	− 0.43	1.48	0.283
OHS Post	Age	− 0.01	− 0.24	0.21	0.909
	Gender	− 0.67	− 5.16	3.81	0.769
	BMI	0.08	− 0.27	0.43	0.652
	Δ SS (stand-sit)	− 0.35	− 0.50	− 0.20	**0.000**

Adjustments for every variable (in blue) were made for the effects of age, gender, and BMI on the postoperative clinical outcomes. Higher BMI values were associated with poorer postoperative PROMs. The table includes only those correlations that reached statistical significance. ΔSS: delta Sacral Slope, difference between standing and sitting SS

## Discussion

### Findings

The main finding of the study was that individualized component positioning achieved through FCPA cannot eliminate the risk of impingement during untested ADLs. Anyway, in more than 50% of patients, no impingement was detected during the evaluated motor tasks. This finding is consistent with the results by Dennis et al. (2023) showing that the prevalence of prosthetic impingement was significantly less in the patient-individualized acetabular component position [2].

Despite being a virtual and theoretical system, FCPA allows for the preoperative identification and, where feasible, elimination of simulated impingement. Consequently, it can be reasonably stated that FCPA contributes to a reduction in impingement risk.

Nevertheless, contrary to our hypothesis, the reduction of prosthetic and bone impingement didn’t lead to a significant improvement in PROMs. Excellent PROMs were achieved regardless of the presence of impingement.

### Hip-spine relationship

Pelvic motion changes the 3D orientation of the acetabulum as patients mobilize [2]. Previous studies have demonstrated that for each one degree of change in sagittal pelvic tilt, a 0.7–0.8 change is incurred in acetabular component anteversion. As the pelvis rotates posteriorly, the functional anteversion of the socket increases, which reduces the risk of anterior impingement and posterior dislocation. Conversely, when the pelvis rotates anteriorly, functional anteversion lessens, reducing the risk of posterior impingement and anterior dislocation [2, 12].

An accurate plan via 3D FCPA helps in preventing impingement and might be the safest way to prevent dislocation and improve long-term outcomes. Recently, a new functional safe zone based on spinopelvic mobility has been proposed by O’Connor [9], with a CoV target from 30° to 50° with the pelvis in a standing position, and from 45° to 65° with the pelvis in a sitting position. In this cohort, only 66 patients (66%) had FCPA-determined implant positioning within the O’Connor safe zone. Therefore, as the safe zone concept might not be suitable to prevent impingement, a patient-specific approach is needed. Consensus of the recent literature shows that there is no common optimal cup position for all patients due to variations in spinopelvic anatomy; hence spine helps dictate the individual’s safe zone [2, 9, 12].

### Motor tasks and impingement

Impingement is related to many factors, the most important are CoV, global offset, extra-articular bone morphology, and soft tissues [26]. Prosthesis-on-prosthesis impingement has been reported to be influenced by implant design and intraoperative implant positioning [20, 27]. Whereas, BB impingement has been reported to be affected by variation in the offset of the implant and in bone geometries [2, 27].

In this series, posterior impingement during hip extension was the most frequently observed type of prosthetic impingement, occurring in 36 hips (36%). Notably, in 7 cases (7%) where initial PB contact was present, impingement evolved into PP impingement following osteophyte removal. Prosthesis-on-prosthesis impingement is considered the primary cause of anterior dislocation related to impingement [28]. We reported three cases in which anterior pelvic tilt occurred during the sit-to-stand transition (SS difference: − 9, − 2, and − 2). In each of these cases, the SS remained greater than 25°, thereby classifying them within the “stuck standing” category [29]. Although this condition may theoretically increase the risk of anterior impingement, no impingement was detected during any of the analyzed motor tasks in these three patients.

### Patient-reported outcome measures (PROMs)

Overall, excellent PROMs were observed in all patients, with only 12 cases (12%) failing to meet the PASS [21, 24, 30]. Giesinger et al. stated that the median FJS-12 was 87.5 [30] while in the present series was 94.

A statistically significant association was identified between BMI and PROMs at the 2-year follow-up, and, specifically, higher BMI values were associated with lower PROMs. While this result is in accordance with several studies [31, 32], other studies do not corroborate these findings [33].

In the multivariate models, a statistically significant correlation was found between postoperative OHS and FJS-12 and the delta SS (standing-sitting) (*P* < 0.000). This result suggests that as the delta SS increases, PROMs tend to decrease. In other words, patients with a pre-existing rigid spine benefit more from THA. This may be explained by the fact that, even in the presence of a rigid spine, restoring good hip mobility allows patients to perform daily activities effectively by relying on hip movement (“hip users”), leading to higher satisfaction. In addition, for patients with hip-spine syndrome, improvements in both hip and back pain have been demonstrated after THA [7]. The decreased anterior tilt of the two lowest lumbar vertebrae and the corresponding loss of lumbar lordosis may reduce facet loading during the stance phase of gait after THA in a stiff spine. This change in lumbar spine kinematics during gait is a potential mechanism to explain the observed improvements in back pain and disability after THA [7].

### Limitations

Several limitations should be acknowledged when concluding the present study. Due to the observational and retrospective design of the study, there was a potential risk of selection and recall bias. Another limitation is the relatively small sample size. This factor is particularly relevant, as it prevented the execution of a conclusive power analysis, precisely due to the limited number of subjects. Moreover, the absence of a control group in which FPCA was not utilized limits the ability to determine whether access to this technology leads to improved PROMs and reduced impingement. Furthermore, the proprietary nature of the software may hinder the reproducibility of the study.

Another limitation was that spinopelvic mobility changes post-RTHA were not considered [34]. In addition, the 3D-CT impingement detection algorithm assessed osseous and prosthetic impingement. However, the presence of soft tissue-related impingement was not addressed [2, 9].

Furthermore, this study relies on pelvic movements in the sagittal plane. Any coronal or axial rotation of the pelvis in functional positions would also affect the acetabular orientation [2]. Another limitation was that the motor tasks analysed may not replicate thoroughly how patients move during ADL, as they do not encompass the full spectrum of movements that can lead to hip dislocation. Finally, the positive effects of 3D functional simulation on the final components’ position rely on robotic accuracy. However, the precision and reproducibility of robotic systems in hip arthroplasty have already been reported [35].

## Conclusions

Individualized component positioning, even when aided by 3D FCPA, does not eliminate the risk of impingement. The concept of a universal"safe zone"may be inadequate to prevent impingement, emphasizing the need for patient-specific implant positioning strategies based on spinopelvic mobility. While impingement did not significantly impact PROMs, optimizing implant positioning through robotic-assisted techniques may contribute to improved long-term outcomes and reduced dislocation risk.

## Data Availability

The data that support the findings of this study are available from the corresponding author upon reasonable request.

## Abbreviations

ADL: Activities of Daily Living
BB: Bone-on-Bone Impingement
BMI: Body Mass Index
CI: Confidence Interval
CoV: Combined Anteversion
CT: Computed Tomography
FCPA: Functional Component Positioning Assessment
FJS-12: Forgotten Joint Score
HOOS: Hip Disability and Osteoarthritis Outcome Score
MD: Mean Difference
OHS: Oxford Hip Score
OR: Odds Ratio
PASS: Patient-Acceptable Symptom State
PB: Prosthesis-on-Bone Impingement
PP: Prosthesis-on-Prosthesis Impingement
PROMs: Patient-Reported Outcome Measures
ROM: Range of Motion
RTHA: Robotic-assisted Total Hip Arthroplasty
SD: Standard Deviation
SS: Sacral Slope
THA: Total Hip Arthroplasty
3D: Three Dimensional
